# Effect of Serum Level of Vitamin D on External Apical Root Resorption in Maxillary Anterior Teeth in Patients under Fixed Orthodontic Treatment

**DOI:** 10.1155/2022/7942998

**Published:** 2022-09-27

**Authors:** Fatemeh Azizi, Nasim Karami, Amin Golshah, Mohammad Moslem Imani, Roya Safari-Faramani

**Affiliations:** ^1^Department of Orthodontic, School of Dentistry, Kermanshah University of Medical Sciences, Kermanshah 6715847141, Iran; ^2^Student Research Committee, School of Dentistry, Kermanshah University of Medical Sciences, Kermanshah 6715847141, Iran; ^3^Social Development and Health Promotion Research Center, Kermanshah University of Medical Sciences, Kermanshah 6715847141, Iran

## Abstract

**Introduction:**

This study aimed to assess the effect of serum level of vitamin D on external apical root resorption (EARR) in maxillary anterior teeth in patients under fixed orthodontic treatment.

**Materials and Methods:**

This retrospective cohort was conducted on patients under fixed orthodontic treatment who were between 12 to 30 years of age. All patients underwent the same treatment technique by the same orthodontist using a 0.022 MBT system. EARR in maxillary anterior teeth was evaluated on pre- and postoperative panoramic radiographs. Blood samples were also collected from patients, and their serum level of vitamin D was measured after the completion of treatment. Data were analyzed by independent *t*-test and Chi-square test (alpha = 0.05).

**Results:**

A reduction in root length was noted in all patients, which was significant (*P* < 0.0001); 75% of patients showed EARR in at least one maxillary incisor. EARR had no significant correlation with the serum level of vitamin D (*P*=0.423).

**Conclusions:**

Serum level of vitamin D had no significant correlation with the occurrence of EARR. However, the high prevalence of EARR calls for measures to minimize it.

## 1. Introduction

Root resorption defect is defined as resorption of the root surface for reasons not related to dental caries or root fractures. External root resorption starts from the outermost surface of the root [1]. Either physiological or pathological, root resorption refers to the loss of tooth structure by the activity of osteoclasts. Pathological root resorption may occur due to a number of reasons such as orthodontic treatment, and traumatic dental injuries [2]. In case of the occurrence of external apical root resorption (EARR), reconstruction of the lost tooth structure is almost impossible. The best strategy to prevent EARR is to control the biomechanical factors involved in its occurrence [3].

Despite the extensive body of the animal and human research, the exact mechanism of root resorption following orthodontic treatment has yet to be fully understood [4, 5]. However, it is assumed that the biological response to long-term controlled mechanical orthodontic forces that cause pressure and tension sites in the periodontium, and the generation of proinflammatory mediators such as prostaglandins and leukotrienes not only cause movement of tooth sockets [6] but also lead to cementum loss and root resorption [7].

EARR that occurs after orthodontic treatment may compromise the treatment's success. This type of root resorption is destructive and irreversible [1]. The diagnosis and follow-up of EARR can be performed by assessment of root length and noticing root shortening on radiographs [8]. Orthodontically induced EARR often occurs in teeth under strong orthodontic forces for a long period of time. It may also occur due to incorrect direction of load application, or in teeth with poor periodontal support [9, 10]. A number of factors are involved in primary root resorption and its progression during the course of orthodontic treatment, which can be categorized as biological factors, mechanical factors, and both [11]. A long course of orthodontic treatment, age, and sex of the patient, type of applied orthodontic force, the direction of tooth movement, type of orthodontic appliance, technique of treatment, abnormal root shape, race, and systemic diseases are among the factors implicated in the occurrence of EARR [5, 12–14].

Absorption, transfer, and deposition of calcium and phosphorus (to a lower extent) in the process of bone mineralization are among the important biological tasks of vitamin D. Serum level of vitamin D is an important parameter in the formation and resorption of hard tissues such as bone and teeth [15]. A previous study found an association between vitamin D receptor gene polymorphism and root resorption [16]. However, a search of the literature by the authors yielded only one study on the role of serum level of vitamin D in the development of EARR, which found no significant correlation [17]. Considering the gap in information on this topic, this study aimed to assess the effect of serum level of vitamin D on EARR in maxillary anterior teeth in patients under fixed orthodontic treatment.

## 2. Materials and Methods

This retrospective cohort study was conducted on patients under fixed orthodontic treatment presenting to a private orthodontic office in Kermanshah city. The study was approved by the ethics committee of Kermanshah University of Medical Sciences (IR.KUMS.REC.1398.387).

The sample size was calculated to be 50 patients according to a previous study [18] assuming alpha = 0.05, and an accuracy of 0.131.

The inclusion criteria were age between 12 to 30 years, having high-quality pre- and postoperative digital panoramic radiographs, and optimal periodontal health prior to initiation of orthodontic treatment (normal gingival tissue, no bleeding on probing). The exclusion criteria were history of systemic diseases, long-term medication intake, history of trauma to the mouth or nose, genetic disorders, maxillofacial developmental disorders, history of oral habits or parafunctions, presence of an impacted canine, history of maxillary orthodontic treatment, history of root resorption prior to orthodontic treatment onset, crown fracture, wear of incisal edges, abnormal roots (very narrow or malformed roots), radiographic evidence of incomplete root formation, and periodontal disease. Also, poor-quality radiographs and those with distortion due to the protrusion of anterior teeth or superimposition were excluded.

The six maxillary anterior teeth were evaluated (a total of 80 teeth). Class of malocclusion, type of orthodontic treatment plan (extraction or nonextraction), number of endodontically treated teeth, and duration of orthodontic treatment were extracted from patient records. All orthodontic treatments had been performed by an experienced and skillful orthodontist with the same technique using a 0.022 slot MBT system.

After obtaining written informed consent from the patients, blood samples were collected from them, and their serum level of vitamin D was measured after the completion of treatment. Blood samples were collected from the participants in a reliable laboratory. Blood samples were collected in sterile tubes under aseptic conditions and centrifuged at 3000 rpm for 5 minutes. The serum level of vitamin D (25-hydroxyvitamin D) was measured by liquid chromatography-tandem mass spectrometry (LC-MS/MS) [19].

The amount of EARR was quantified on pre- and postoperative panoramic radiographs. Postoperative panoramic radiographs were obtained with the same condition as the preoperative radiographs (in terms of exposure parameters, standard position, and taking the radiograph by the same operator). All radiographs had optimal quality and resolution at the site of respective teeth, and adequate density and contrast for detection of EARR.

To assess and quantify EARR, first, the tooth length was measured at the longitudinal axis, which follows the canal path from the apex to the incisal edge of the anterior teeth, perpendicular to the cementoenamel junction, by a digital caliper with 0.01 mm accuracy. Any change in tooth size on the postoperative radiograph compared with the preoperative radiograph (which could have occurred due to changed distance between the X-ray tube and tooth, or film displacement) was adjusted by using the numerical value of crown length (was assumed to remain constant during the study period) and the correction coefficient (calculated by dividing the crown length on the preoperative radiograph by the crown length on the postoperative radiograph). Accordingly, the actual amount of EARR was calculated using the following formula:

The numerical value of EARR = (root length after treatment × correction coefficient) − root length prior to treatment.

In this study, root resorption was the outcome variable, and patients with > 2 mm root resorption [3] were considered to have EARR.

The Kolmogorov-Smirnov test was used to assess the normality of data distribution. Data were analyzed by independent *t*-test and Chi-square test, whenever appropriate. All statistical analyses were carried out using STATA software at a 0.05 level of significance.

## 3. Results

Table 1 presents the mean root length of maxillary anterior teeth before and after orthodontic treatment. As shown, root length decreased in all teeth after treatment, and this reduction was statistically significant (*P* < 0.0001 for all teeth). Accordingly, 75% of the patients (*n* = 60 patients) had EARR in at least one of their maxillary incisors.

Table 2 Reports the frequency of EARR in maxillary anterior teeth of patients based on the type of malocclusion. The results showed no significant correlation between the class of malocclusion and EARR (Chi statistics = 1.127, *P*=0.569). However, a higher percentage of class III patients experienced EARR (84%).

Table 3 presents the frequency of EARR in patients based on serum level of vitamin D, orthodontic treatment plan (extraction versus nonextraction), and endodontic treatment of teeth. The correlation between serum level of vitamin D and EARR was not significant (Chi statistics = 0.2402, *P*=0.423). Despite the slightly higher frequency of EARR in cases with an extraction treatment plan, this difference was not significant (Chi statistics = 0.2241, *P*=0.636). Also, despite the slightly higher frequency of EARR in teeth with endodontic treatment, the correlation between EARR and endodontic treatment was not significant (Chi statistics = 0.2402, *P*=0.624).

Duration of treatment was slightly higher in patients with EARR (761 ± 198.74 days versus 707 ± 231.63 days) but this difference was not significant either (*P*=0.316).

## 4. Discussion

Orthodontics is the only dental field that uses the inflammatory process to solve esthetic and functional dental problems. However, some degrees of root resorption are inevitable in the course of orthodontic treatment [20]. This study assessed the effect of serum level of vitamin D on external apical root resorption in maxillary anterior teeth in patients under fixed orthodontic treatment. The results showed a significant reduction in root length following orthodontic treatment in all cases. Of 80 patients, 75% (*n* = 60) showed EARR in at least one of their maxillary incisors. One possible reason for the reduction in root length may be the protrusion of teeth, especially in nonextraction orthodontic treatment plans.

Salehi et al. [21] assessed root resorption in maxillary anterior teeth of patients with vertical malocclusion after fixed orthodontic treatment and showed that all patients experienced EARR in at least one tooth. The prevalence of EARR in their study was higher than that in the present study, which may be due to racial, age, and gender differences between the study populations or different methodologies, sampling, and criteria used to define EARR. Ravanmehr et al. [22] evaluated the effect of fixed orthodontic treatment on the EARR of maxillary incisors and reported EARR in 73.7% of the patients, which was highly similar to the rate found in the present study.

Considering the high prevalence of EARR, it is important to find strategies to minimize its occurrence and detect patients more susceptible to EARR [23]. Accordingly, the serum level of vitamin D was measured and its correlation with EARR was analyzed in the present study. The results showed no significant correlation between EARR and serum level of vitamin D. Fontana et al. [16] evaluated the DNA of class II division I patients under fixed orthodontic treatment for assessment of vitamin D receptor gene polymorphisms and their association with EARR and found that vitamin D receptor TaqI polymorphism was associated with EARR in orthodontic patients. Tehranchi et al. [17] assessed the correlation between the serum level of vitamin D and the occurrence of EARR. They reported a Pearson's correlation coefficient of 0.15. After applying a linear regression model to control for the effect of confounders, they found no significant correlation between serum levels of vitamin D and EARR, which was in line with the present findings. In a recent study, Al-Attar and Abid [24] evaluated the serum level of vitamin D3 and detected no significant correlation between the serum level of vitamin D and root resorption due to orthodontic treatment. However, it should be noted that they only evaluated patients for a 3-month period at the onset of treatment during the leveling and alignment phase, and thus, their results cannot reflect the effect of the entire course of orthodontic treatment; whereas, the present study evaluated pre- and post-treatment radiographs of patients who underwent comprehensive orthodontic treatment. Booij-Vrieling et al. [25] in an animal study found no significant correlation between serum levels of vitamin D and EARR either.

However, Seifi et al. [26] discussed that reduction in serum level of vitamin D is one of the important factors in the occurrence of EARR, and vitamin D can have a strong supportive role in the prevention of EARR. Also, Kawakami and Takano-Yamamoto [27] demonstrated that vitamin D increased the regeneration of tooth-supporting structures, particularly the alveolar bone after orthodontic treatment. The process of resorption includes complex interactions between the inflammatory cells, cytokines, enzymes, and clast cells. The metabolic activity of vitamin D includes indirect stimulation of osteoclastogenesis by regulation of expression of some secondary messengers [28]. Since this active metabolite is considered a regulator in the formation and activity of osteoclasts and odontoclasts in regenerative procedures, it is assumed that vitamin D may have an indirect role in the pathophysiology of regeneration [25].

A small sample size due to the COVID-19 pandemic was a limitation of this study. Future multicenter studies with a larger sample size are required to better elucidate this topic.

## 5. Conclusion

Serum level of vitamin D had no significant correlation with the occurrence of EARR. However, the high prevalence of EARR calls for measures to minimize it.

## Figures and Tables

**Table 1:** Mean root length of maxillary anterior teeth before and after orthodontic treatment (mm).

Tooth	Number	Mean root length preoperatively (mean and std. deviation)	Mean root length postoperatively (mean and std. deviation)	*P*-value
Right canine	79	27.06 ± 2.33	25.49 ± 2.31	< 0.0001
Right lateral incisor	78	22.84 ± 2.20	20.99 ± 2.16	< 0.0001
Right central incisor	80	23.75 ± 1.95	21.88 ± 1.98	< 0.0001
Left central incisor	80	23.76 ± 1.88	22.03 ± 2.09	< 0.0001
Left lateral incisor	78	22.86 ± 1.98	21.08 ± 1.99	< 0.0001
Left canine	80	27.07 ± 2.25	25.41 ± 2.13	< 0.0001

**Table 2:** Frequency of EARR in maxillary anterior teeth of patients based on the type of malocclusion (number of patients is reported).

Malocclusion type	EARR		Total patients (%)
	Absent (%)	Present (%)	
I	7 (28)	18 (72)	25 (100)

II	10 (28)	26 (72)	36 (100)

III	3 (16)	16 (84)	19 (100)

Total	20 (25)	60 (75)	80 (100)

**Table 3:** Frequency of EARR in patients based on serum level of vitamin D, orthodontic treatment plan (extraction versus nonextraction), and endodontic treatment of teeth.

Variable	Categories	Absence of EARR (%)	Presence of EARR (%)	Total (%)
Serum level of vitamin D	Adequate	7 (20)	28 (80)	35 (100)
	Inadequate	11 (32)	23 (68)	34 (100)
	Deficiency	2 (18)	9 (82)	11 (100)
	Total	20 (25)	60 (75)	80 (100)

Treatment plan	Extraction	15 (24)	48 (76)	63 (100)
	Nonextraction	5 (29)	12 (71)	17 (100)
	Total	20 (25)	60 (75)	80 (100)

History of endodontic treatment	Absent	19 (26)	55 (74)	74 (100)
	Present	1 (17)	5 (83)	6 (100)
	Total	20 (25)	60 (75)	80 (100)

## Data Availability

The data used to support the findings of this study were supplied by Amin Golshah under license and data will be available on request. Requests for access to these data should be made to Amin Golshah.
